# Genetic Polymorphism of Cancer Susceptibility Genes and HPV Infection in Cervical Carcinogenesis

**DOI:** 10.4061/2011/364069

**Published:** 2011-05-31

**Authors:** Osamu Nunobiki, Masatsugu Ueda, Eisaku Toji, Michiko Yamamoto, Kyoko Akashi, Naomi Sato, Shinji Izuma, Kiyo Torii, Ichiro Tanaka, Yoshiaki Okamoto, Sadamu Noda

**Affiliations:** ^1^Department of Medical Technology, Kobe Tokiwa University, 6-2 2 chome, Ohtanicho, Nagataku, Hyogo, Kobe 653-0838, Japan; ^2^Department of Cytopathology and Gynecology, Osaka Cancer Prevention and Detection Center, Osaka 536-8588, Japan; ^3^Department of Obstetrics and Gynecology, Japanese Red Cross Kyoto Daiichi Hospital, Kyoto 605-0981, Japan

## Abstract

It is widely accepted that specific human papillomavirus (HPV) types are the central etiologic agent of cervical carcinogenesis. However, a number of infected women do not develop invasive lesions, suggesting that other environmental and host factors may play decisive roles in the persistence of HPV infection and further malignant conversion of cervical epithelium. Although many previous reports have focused on HPV and environmental factors, the role of host susceptibility to cervical carcinogenesis is largely unknown. Here, we review the findings of genetic association studies in cervical carcinogenesis with special reference to polymorphisms of glutathione-S-transferase (GST) isoforms, p53 codon 72, murine double-minute 2 homolog (MDM2) gene promoter 309, and FAS gene promoter -670 together with HPV types including our recent research results.

## 1. Introduction

Cervical cancer is the second most common cancer in women worldwide, and is both a preventable and a curable disease especially if identified at an early stage. It is widely accepted that specific human papillomavirus (HPV) types are the central etiologic agent of cervical carcinogenesis. Other environmental and host factors also play decisive roles in the persistence of HPV infection and further malignant conversion of cervical epithelium [1]. Although many previous reports have focused on HPV environmental factors, the role of host susceptibility to cervical carcinogenesis is largely unknown.

A large number of previous studies have suggested the possible correlation between genetic polymorphisms of cancer susceptibility genes and the higher risk of human malignant tumors [2, 3]. Genetic studies lead to a true association are expected to increase understanding of the pathogenesis of each malignancy and to be a powerful tool of prevention and prognosis in the future. Here, we review the findings of genetic polymorphisms of several cancer susceptibility genes together with HPV types in cervical carcinogenesis based on our recent research results using exfoliated cervical cell samples or human cervical squamous carcinoma cell lines. Our studies were approved by our institutional ethics committee, and all samples were obtained with informed consent. To compare the polymorphic features of each genotype and HPV status between normal, LSIL, and HSIL groups, Fisher's exact test or Pearson's chi-square test was used. A level of *P* < .05 was accepted as statistically significant.

## 2. Glutathione-S-Transferase GSTM1, GSTT1 Polymorphisms

The genes of glutathione-S-transferase (GST) family encode enzymes that appear to be critical in cellular protection against the cytotoxic effects. GSTs play an important role in conjugating glutathione to the products of endogenous lipid peroxidation and inactivating organic hydroperoxides via selenium-independent glutathione peroxidase activity, thus protecting the cell from the deleterious effects of oxidative stress [4]. GST isoforms GSTM1 and GSTT1 gene deletions may promote the development of cervical dysplasia by moderating the activation and detoxification of polycyclic hydrocarbons and other compounds that influence oxidative stress and DNA adduct formation [5].

We conducted GST genotype analysis together with HPV typing in a total of 198 cervical smear samples obtained from the patients who received cervical cancer screening. They consist of 54 normal, 102 low-grade squamous intraepithelial lesion (LSIL), and 42 high-grade SIL (HSIL). The protocol of this study was approved by our institutional review board, and all samples were obtained from Japanese women with informed consent. The exfoliated cervical cells were disrupted with lysis buffer, and genomic DNA was extracted with phenol-chloroform and precipitated with ethanol using standard techniques. The GSTM1 and GSTT1 genetic polymorphisms were evaluated using multiplex PCR techniques according to the method reported by Chen et al. [6] with some modifications as previously described [2]. The presence of various HPV types was examined by L1-PCR system using published consensus primers (L1C1 and L1C2) [7] according to the method reported by Nagano et al. [8].

**Figure 1:**
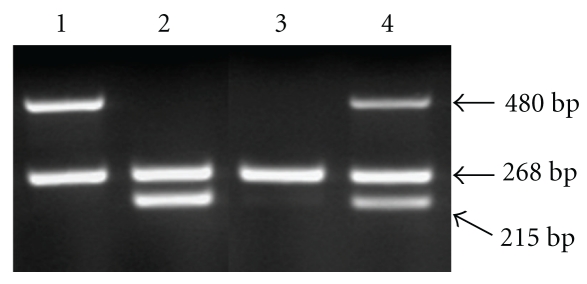
Genotyping of GSTM1 and GSTT1 by multiplex PCR. Lane 1: null GSTM1 genotype (absence of 215 bp fragment). Lane 2: null GSTT1 genotype (absence of 480 bp fragment). Lane 3: null GSTM1 and GSTT1 genotypes (absence of 215 and 480 bp fragments). Lane 4: present GSTM1 and GSTT1 genotypes. *β*-globin as a positive control is detected as 268 bp fragment.

Figure 1 shows an example for genotyping of GSTM1 and GSTT1. The polymorphic deletion of the GSTM1 and GSTT1 genes was determined by multiplex PCR. The absence of 215 or 480 bp fragment indicated null GSTM1 or GSTT1 genotype, respectively.  Table 1 shows frequency of high-risk HPV and GSTM1, GSTT1 polymorphisms in 198 exfoliated cervical cell samples examined. The 42 patients with HSIL had significantly higher frequency of high-risk HPV than 102 with LSIL and 54 controls. There was no significant difference in the frequency of null GSTM1 genotype between SILs and controls, whereas the 42 patients with HSIL had statistically higher frequency of null GSTT1 genotype than 102 with LSIL and 54 controls. As shown in Table 2, the 31 patients with HSIL had also statistically higher frequency of null GSTT1 genotype than 28 with LSIL among the 69 patients with high-risk HPV.

Previous epidemiological studies of GST and cervical neoplasia found no significant differences in the frequency of GSTM1 or GSTT1 in women with SIL or cancer compared to controls with normal cervical pathology [5, 9, 10]. In our investigation using exfoliated cervical cell samples from a Japanese population, the GSTT1 null genotype was more common among HSIL cases than LSIL cases and controls. Moreover, the patients with HSIL also had higher frequency of null GSTT1 genotype than those with LSIL among high-risk HPV group. GSTT1 differs from other classes of GSTs in its lack of activity towards the GST model substrate 1-chloro-2,4-dinitrobenzene and its failure to bind to S-hexyl-glutathione affinity matrices [11]. The gene defect of GSTT1 was reported to be associated with an increased risk of myelodysplastic syndromes [12], astrocytoma, and meningioma [13]. We previously examined GSTM1 and GSTT1 genotypes in 104 cell lines originating from a variety of human malignant tumors and found that GSTT1 null genotype was more common in cervical cancer cells [2]. It might be of interest to further examine the difference in the polymorphic frequency of the null GSTT1 genotype between SILs and invasive cervical cancer to clarify whether this genotype alteration occurs prior to the development of malignant phenotype cells or late in the development of neoplastic cells.

## 3. p53 Codon 72 Polymorphism

p53 is a tumor suppressor gene involved in multiple pathways including apoptosis, cellular transcriptional control, and cell cycle regulation [14, 15]. A large number of human tumors, including smoke-induced lung cancer, show mutations and deletions of the p53 gene that result in loss of tumor suppression function and cell cycle deregulation [16]. A polymorphism at codon 72 of the p53 gene results in the substitution of arginine (Arg) for proline (Pro) in the gene product. It has been suggested that the homozygous Arg genotype increased the susceptibility of p53 protein to degradation by E6 protein derived from oncogenic HPV [17].

We conducted genotype analysis of p53 codon 72 together with HPV typing in a total of 198 cervical smear samples obtained from the patients who received cervical cancer screening. They consist of 54 normal, 102 LSIL, and 42 HSIL, as described above. PCR restriction fragment length polymorphism (RFLP) analysis of codon 72 of the p53 gene, which was modified from a technique described by Ara et al. [18], was conducted to identify p53 genotypes. HPV types were examined using L1-PCR, as describer above.

**Figure 2:**
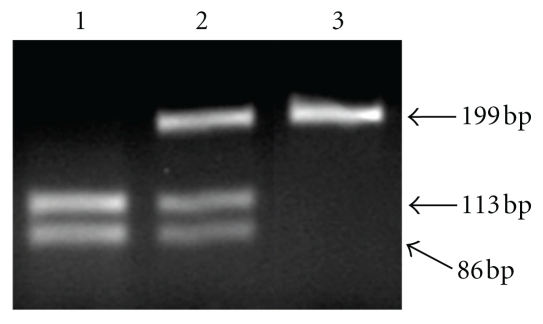
Genotyping of p53 codon 72 by PCR-RFLP. Lane 1: Arg/Arg homozygote. Lane 2: Arg/Pro heterozygote. Lane 3: Pro/Pro homozygote. The fragment of 199 bp is the nondigested PCR product from the Pro allele. Fragments of 113 and 86 bp result from BstUI digestion of the Arg allele.

As shown in Figure 2, the Arg allele is cleaved by BstUI and yields two small fragments (113 and 86 bp). The Pro allele is not cleaved by BstUI and has a single 199 bp band. The heterozygote contains three bands (199, 113, and 86 bp).  Table 3 shows HPV status and polymorphic frequency of p53 codon 72 in 198 samples examined. The 42 patients with HSIL had significantly higher frequency of high-risk HPV than 102 with LSIL and 54 controls, as described above. The differences in the polymorphic frequency of p53 Arg, Arg/Pro, and Pro genotypes between SILs and controls were statistically not significant. When the Arg genotype was compared to the Arg/Pro + Pro genotypes, there was again no statistical difference in the genotype prevalence between SILs and controls with or without high-risk HPV, as shown in Table 4.

Our present results revealed that the differences in the polymorphic frequency of p53 Arg, Arg/Pro, and Pro genotypes between SILs and controls were statistically not significant. Moreover, neither Arg nor Pro allele affected the increased risk of SILs with or without high-risk HPVs compared to controls. Some previous studies have reported no correlation between germline polymorphisms of the p53 codon 72 and increased risk of cervical cancer [19–21]. The other study reported by Nishikawa et al. [22] using cervical condyloma, dysplasia, and cancer tissue samples demonstrated that no statistically significant differences in the distribution of p53 genotypes were found among the patients with these diseases, regardless of HPV status. The recent meta-analysis on p53 codon 72 polymorphism reported by Klug et al. [23] also demonstrated that no statistically significant differences in the distribution of p53 genotypes were found among the patients with cervical diseases. These data suggest that the p53 codon 72 polymorphism is unlikely to be associated with the development of HPV-associated cervical neoplasms.

## 4. MDM2-SNP309

Murine double-minute 2 homolog (MDM2) is the key negative regulator of p53, and dysfunction of these genes may be associated with an increased rate of accumulation of genetic errors, thereby enhancing the progression of the disease. A single nucleotide polymorphism (SNP) in the MDM2 gene promoter, SNP309 (a T to G change at nucleotide 309 in the first intron), increases the affinity of the promoter for the transcription activator Sp1, resulting in higher level of MDM2 mRNA and MDM2 protein and a subsequent attenuation of the p53 pathway [24]. SNP309 occurs at a relatively high frequency in the general population and has been shown to be associated with accelerated tumorigenesis and the timing of cancer onset [24–27]. However, there have been very few reports on the correlation between SNP of MDM2 gene and cervical cancer susceptibility [28].

We conducted genotype analysis of MDM2-SNP309 together with HPV typing in a total of 195 cervical smear samples obtained from patients with consent who received cervical cancer screening. They consist of 52 normal, 102 LSIL, and 41 HSIL. Eight human cervical squamous carcinoma cell lines (SKG-I, SKG-II, SKG-IIIa, SKG-IIIb, OMC-1, YUMOTO, QG-U, and QG-H) were also used for genotype analysis of MDM2-SNP309 together with HPV typing. All cell lines were originating from Japanese women, as previously described [2]. For MDM2-SNP309 genotyping, two independent PCR assays for each allele, modified from a technique described by Menin et al. [25], were performed using primer pairs specific for the two alleles. HPV types were examined using L1-PCR, as describer above.

**Figure 3:**
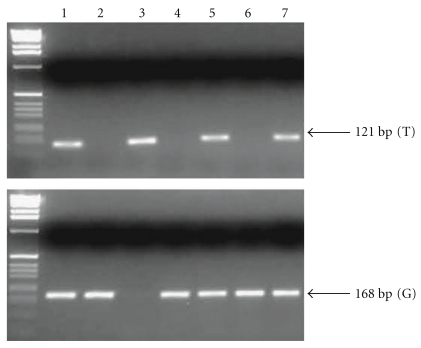
Representative genotyping of MDM2-SNP309 by two independent PCR assays for each allele. Lanes 1, 5, and 7: TG heterozygote. Lanes 2, 4, and 6: GG homozygote. Lane 3: TT homozygote. The wild-type (T) and the mutant (G) allele yields 121-bp and 168-bp fragments, respectively.

**Figure 4:**
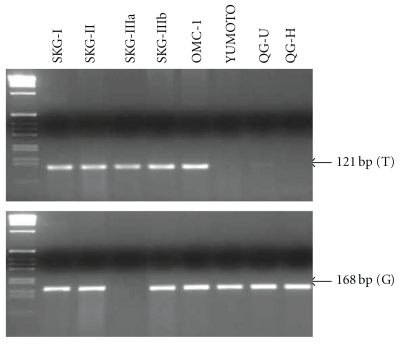
Genotyping of MDM2-SNP309 in 8 cervical squamous carcinoma cell lines by two independent PCR assays for each allele. The TT genotype was detected only for SKG-IIIa, whereas the TG genotype for SKG-I, SKG-II, SKG-IIIb, and OMC-1, and the GG genotype for YUMOTO, QG-U, and QG-H cell lines, respectively.

Figure 3 shows a representative genotyping of MDM-SNP309 by two independent PCR assays. The wild-type (T) and the mutant (G) allele yield 121-bp and 168-bp fragment, respectively.  Table 5 shows the frequency of high-risk HPV and MDM2-SNP309 in 195 exfoliated cervical cell samples examined. When TT genotype was compared to TG + GG genotype, 41 patients with HSIL had significantly higher frequency of high-risk HPV than 102 with LSIL and 52 controls; however, there were no statistical significant differences in the TG + GG genotype prevalence and allele frequencies between SILs and controls. No statistical difference was also found in the genotype frequency of MDM2-SNP 309 between SILs and controls among 127 patients without high-risk HPV. However, there was an increased OR for TG + GG genotype in HSIL cases compared to controls among 68 patients with high-risk HPV, as shown in Table 6. Interestingly, 21 cases with HPV types 16 and/or 18 had significantly higher frequency of the TG + GG genotype and G allele than 47 with other types of high-risk HPV, as indicated in Table 7. Moreover, as shown in Figure 4, genotyping of MDM2-SNP309 in 8 cervical squamous carcinoma cell lines revealed that TT genotype was detected only in the SKG-IIIa cell line, whereas the other 7 of 8 (87.5 %) cell lines had TG or GG genotype. In addition, 7 of 8 cell lines except for YUMOTO were positive for high-risk HPV.

Recently, Meissner Rde et al. [28] tested the hypothesis that this functional variant in the MDM2 promoter was associated with either risk or early age diagnosis of cervical cancer in a Brazilian population. A primer-introduced restriction analysis PCR assay was used to genotype the MDM2-SNP309 of 72 cervical carcinoma patients and 100 healthy women. However, no statistically significant association was observed between SNP309 and cervical cancer. Moreover, they could not find allele or genotype frequency differences between the group of patients with cancer diagnosis at an early age (younger than 40 years old) and the group of older patients. In contrast, Arvanitis and Spandidos [29] demonstrated that MDM2 was one of the potential candidates for the development of cervical neoplasms. They analyzed the mRNA expression profiles of 24 G1/S checkpoint genes in cancer and SIL of the uterine cervix. In total 35 squamous cervical carcinomas, 26 HSIL, 33 LSIL tissues, and 28 normal uterine cervix specimens as controls were assessed by RT-PCR. MDM2 was found to be upregulated in SIL, while RBL1 was found to be downregulated in all three groups of cases.

Our present results using exfoliated cervical cell samples demonstrated that there was an increased OR for TG + GG genotype in HSIL cases compared to controls among the patients with high-risk HPV. We observed that HPV types 16 and 18, the most prevalent and aggressive types worldwide, are predominant in cases with TG + GG genotype and G allele. Moreover, 7 of 8 human cervical squamous carcinoma cell lines that possess high-risk HPV except YUMOTO also showed TG or GG genotype. These observations suggest that MDM2-SNP309 and high-risk HPV infection may be cooperatively associated with cervical carcinogenesis. It would be of interest to further evaluate whether MDM2-SNP309 has the potential to be used in conjunction with HPV-DNA testing and cervical cytology for the management of SIL patients.

## 5. Fas Gene Promoter -670 Polymorphism

Apoptosis is a physiological process that regulates normal homeostasis, and alterations of apoptosis-related genes are likely to contribute to the pathogenesis of autoimmune diseases [30] and malignant tumors [31]. Among various cell surface death receptors, Fas/CD95, a transmembrane receptor, is known as a member of tumor necrosis factor (TNF) receptors superfamily [32]. Downregulation of Fas with resultant resistance to death signals has been reported in many cancers [33–35]. The transcriptional expression of Fas gene is regulated by a number of genetic elements located in the 5′ upstream promoter region of the gene. SNP at -670 in the enhancer region (A/G) situates at a binding element of gamma interferon activation signal (GAS). Homozygous for G allele could result in a complete deletion of the binding sequence of transcription element GAS, which is responsible for the signal emanated through STAT1, and in a significant alteration in the gene expression [36, 37]. However, the correlation between this SNP and cancer susceptibility including the risk of gynecological malignancies has not been extensively studied.

We conducted genotype analysis of Fas gene promoter -670 together with HPV typing in a total of 279 cervical smear samples obtained from the patients with consent who received cervical cancer screening. They consist of 63 normal, 167 LSIL, and 49 HSIL. Eight human cervical squamous carcinoma cell lines (SKG-I, SKG-II, SKG-IIIa, SKG-IIIb, OMC-1, YUMOTO, QG-U, and QG-H) were also used for genotype analysis of this SNP together with HPV typing, as described above. PCR-RFLP analysis of the Fas gene promoter -670, modified from a technique described by Lee et al. [38], was conducted, and HPV types were examined using L1-PCR, as describer above.

**Figure 5:**
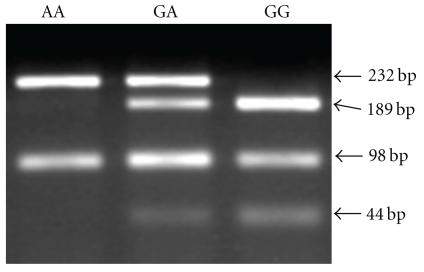
Genotyping of Fas gene promoter -670 in DNA samples from peripheral blood lymphocytes by PCR-RFLP. The genotypes AA (232 bp), GA (189, 233 bp), and GG (189 bp) are shown.

**Figure 6:**
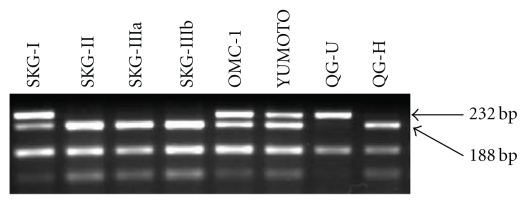
Genotyping of Fas gene promoter -670 in 8 cervical squamous carcinoma cell lines by PCR-RFLP. The AA genotype was detected only for QG-U, whereas the GA genotype for SKG-I, OMC-1, and YUMOTO, and the GG genotype for SKG-II, SKG-IIIa, SKG-IIIb, and QG-H cell lines, respectively.

Figure 5 shows an example for genotyping of Fas gene promoter -670 in exfoliated cervical cell samples. The fragments of 232 and 188 bps indicated the AA and GG genotypes, respectively. The GA genotype contained these two bands.  Table 8 shows the frequency of high-risk HPV and Fas promoter -670 polymorphism in 279 samples examined. When AA genotype was compared to GA + GG genotype, 49 patients with HSIL had significantly higher frequency of high-risk HPV and GA + GG genotype than 167 with LSIL and 63 controls. G allele frequency was also higher in HSIL than in LSIL and controls. There was no statistical difference in the GA + GG genotype prevalence between SILs and controls among 183 patients without high-risk HPV as shown in Table 9. However, there was an increased odds ratio (OR) for GA + GG genotype in HSIL cases compared to controls among 96 patients with high-risk HPV. As shown in Figure 6, genotyping of Fas gene promoter -670 in 8 cervical squamous carcinoma cell lines revealed that AA genotype was detected only in the QG-U cell line, whereas the other 7 of 8 (87.5 %) cell lines had GA or GG genotype.

Polymorphisms in the promoter region or 5′ flanking region of genes can lead to different levels of gene expression and have been also implicated in a number of diseases. Recently, Lai et al. [39] conducted Fas promoter -670 polymorphism analysis using surgical and biopsy tissue specimens of cervical neoplasm and reported that the frequency of A allele and AA genotype increased in accordance with the multistep carcinogenesis from LSIL, HSIL to invasive squamous cell cancer. They stated that A allele and AA genotype, conferring an intact GAS element and more efficient Fas expression, could be one of the mechanism that cells use to avoid carcinogenesis. In contrast, our present results using exfoliated cervical cell samples demonstrated that the frequency of GA + GG genotype or G allele increased from LSIL to HSIL. Moreover, there was an increased OR for GA + GG genotype in HSIL cases compared to controls among the patients with high-risk HPV. Recently, Engelmark et al. [40] and Dybikowska et al. [41] have demonstrated that AA genotype in Fas gene promoter at -670 position may not be engaged in the development of cervical neoplasia in Swedish and Polish population, respectively. These discrepancies may be due to the ethnic variation of genotype frequency of Fas gene promoter in different geographical regions.

 Previous studies [42, 43] have demonstrated that high-risk HPV infection is inversely correlated with apoptosis of cervical epithelial cells and that a decrease of apoptosis is closely associated with higher histologic grade of SIL. In cervical cancer tissues and cell lines, significant decrease in the expression levels of Fas has been also reported [43, 44]. The higher frequency of GA or GG genotype in HSIL cases in our series may result in a significant decrease in Fas gene expression and subsequent escape from apoptosis of the cells in high-risk HPV-related cervical carcinogenesis. Interestingly, 7 of 8 human cervical squamous carcinoma cell lines that possess high-risk HPV except for YUMOTO also showed GA or GG genotype. Fas gene promoter polymorphism may be closely associated with cervical carcinogenesis particularly in high-risk HPV group. These observations are potentially important in managing SIL patients by cytologic examination and in understanding the pathogenesis of cervical cancer.

## 6. Conclusion and Future Directions

Here, we review the findings of genetic association studies in cervical carcinogenesis with special reference to polymorphisms of GST isoforms, p53 codon 72, MDM2-SNP309, and FAS gene promoter -670 together with HPV types including our recent research results. Our studies using exfoliated cervical cell samples or human cervical squamous carcinoma cell lines have demonstrated that the GSTT1 null genotype, the TG/GG genotype of MDM2-SNP309, and the GA/GG genotype or G allele of Fas promoter -670 are closely associated with cervical carcinogenesis together with high-risk HPV infection. It would be of interest to further evaluate whether these polymorphisms could be used as a disease marker for the natural history of cervical neoplasms in a setting of longitudinal cohort study and for the determination of appropriate screening interval in patients with or without high-risk HPV. 

HPV are the etiologic agents of cervical and other epithelial cancers. Persistence of infections by high-risk HPV types is the single greatest risk factor for malignant progression. Vaccination against HPV types 16 and 18 has commenced or will soon commence in a number of countries. Our studies have demonstrated that the frequency of G allele increased from LSIL to HSIL and that there was an increased OR for G allele in HSIL cases with high-risk HPV types including 52 and 58. It is known that geographically different oncogenic HPV types 52 and 58 are more prevalent than 16 and 18 in East Asia. HPV prevalence should be considered and treated individually regarding the strategy that best suits the HPV types in a given geographical area. It is likely that novel strategies in combination with vaccination against HPV types 16 and 18 are required in those countries where other types of HPV may be more prevalent. Moreover, further studies on the differential gene expression profiles between normal cervical keratinocytes and cervical cancer cell lines may provide the better understanding for the effect of these polymorphisms in the sequence of cervical carcinogenesis.

## Figures and Tables

**Table 1:** Frequency of high-risk HPV and GSTM1, GSTT1 polymorphisms in exfoliated cervical cell samples.

Lesions	Number with high-risk HPV	GSTM1null	GSTT1 null
Normal (*n* = 54)	10 (18.5%)^a^	28 (51.9%)	24 (44.4%)^c^
LSIL (*n* = 102)	28 (27.5%)^b^	55 (53.9%)	40 (39.2%)^d^
HSIL (*n* = 42)	31 (73.8%)^a, b^	20 (47.6%)	29 (69.0%)^c, d^

^a^ *P* < .0001, OR = 12.4 *χ* ^2^  versus normal. ^b^ *P* < .0001, OR = 7.4 *χ* ^2^ versus LSIL. ^c^ *P* = .0162, OR = 2.8 *χ* ^2^  versus normal. ^d^ *P* = .0011, OR = 3.5 *χ* ^2^  versus LSIL.

**Table 2:** HPV status and frequency of GSTT1 polymorphism in exfoliated cervical cell samples.

Study group	*n*	GSTT1null
High-risk HPV−		
Normal	44	20 (45.5%)
LSIL	74	31 (41.9%)
HSIL	11	8 (72.7%)

High-risk HPV+		
Normal	10	4 (40.0%)
LSIL	28	9 (32.1%)^a^
HSIL	31	21 (67.7%)^a^

^a^ *P* = .0063, OR = 4.4 *χ* ^2^  versus LSIL.

**Table 3:** Frequency of high-risk HPV and p53 codon 72 polymorphism in exfoliated cervical cell samples.

Lesions	Number with high-risk HPV	Aminoacid at p53 codon 72		
		Arg	Arg/Pro	Pro
Normal (*n* = 54)	10 (18.5%)^a^	24 (44.4%)	23 (42.6%)	7 (13.0%)
LSIL (*n* = 102)	28 (27.5%)^b^	38 (37.3%)	40 (39.2%)	24 (23.5%)
HSIL (*n* = 42)	31 (73.8%)^a, b^	18 (42.9%)	16 (38.1%)	8 (19.0%)

^a^ *P* < .0001, OR = 12.4 *χ* ^2^  versus normal. ^b^ *P* < .0001, OR = 7.4 *χ* ^2^  versus LSIL.

**Table 4:** HPV status and frequency of p53 codon 72 polymorphism in exfoliated cervical cell samples.

Study group	*n*	Aminoacid at p53 codon 72	
		Arg	Arg/Pro + Pro
High-risk HPV−	
Normal	44	20 (45.5%)	24 (54.5%)
LSIL	74	26 (35.1%)	48 (64.9%)
HSIL	11	4 (36.4%)	7 (63.6%)

High-risk HPV+			
Normal	10	4 (40.0%)	6 (60.0%)
LSIL	28	12 (42.9%)	16 (57.1%)
HSIL	31	14 (45.2%)	17 (54.8%)

**Table 5:** Frequency of high-risk HPV and MDM2-SNP309 in exfoliated cervical cell samples.

Lesions	Number with high-risk HPV	Genotype frequency		Allele frequency	
		TT	TG + GG	T	G
Normal (*n* = 52)	10 (19.2%)^a^	11 (21.2%)	41 (78.8%)	49 (47.2%)	55 (52.8%)
LSIL (*n* = 102)	28 (27.5%)^b^	26 (25.4%)	76 (74.6%)	104 (50.6%)	100 (49.4%)
HSIL (*n* = 41)	30 (73.2%)^a, b^	7 (17.1%)	34 (82.9%)	37 (45.1%)	45 (54.9%)

^a^ *P* = .0010  *χ* ^2^  versus normal. ^b^ *P* = .0019  *χ* ^2^  versus LSIL.

**Table 6:** HPV status and frequency of MDM2-SNP309 in exfoliated cervical cell samples.

Study group	*n*	Genotype at MDM2-SNP309		OR	95% CI	*P* value
		TT	TG + GG			
High-risk HPV−						
Normal	42	8 (18.2%)	36 (81.8%)	1		
LSIL	74	14 (18.4%)	60 (81.1%)	0.95	0.36–2.49	.921
HSIL	11	4 (36.7%)	7(63.3%)	0.41	0.10–1.59	.186

High-risk HPV+						
Normal	10	3 (30.0%)	7 (70.0%)	1		
LSIL	28	12 (42.9%)	16 (57.1%)	0.38	0.10–1.42	.475
HSIL	30	3 (10.0%)	27 (90.0%)	8.88	2.34–33.63	.003

OR: odds ratio.

CI: confidence interval.

**Table 7:** High-risk HPV types and frequency of MDM2-SNP309 in exfoliated cervical cell.

Study group	Genotype frequency		Allele frequency	
	TT	TG +	T	G
HPV types 16, 18 (*n* = 21)	2 (9.5%)	19 (90.5%)	14 (33.3%)	28 (66.7%)^b^
HPV other types (*n* = 47)	18 (38.2%)	29 (61.8%)^a^	51 (54.2%)	43 (45.8%)^b^

^a^ *P* = .0161  *χ* ^2^  versus  HPV types 16, 18.

^b^ *P* = .0240  *χ* ^2^  versus  HPV types 16, 18.

**Table 8:** Frequency of high-risk HPV and Fas promoter -670 polymorphism in exfoliated cervical cell samples.

Lesions	Number with high-risk HPV	Genotype frequency		Allele frequency	
		AA	GA + GG	A	G
Normal (*n* = 63)	10 (15.9%)^a ^	19 (30.2%)	44 (69.8%)^c ^	67 (53.2%)	59 (46.8%)^e ^
LSIL (*n* = 167)	46 (27.5%)^b ^	51 (30.5%)	116 (69.5%)^d ^	165 (49.4%)	169 (50.6%)^f ^
HSIL	40 (81.6%)^a, b ^	5 (10.2%)	44 (89.8%)^c, d ^	37 (37.8%)	61 (62.2%)^e, f ^

^a^ *P* < .0001*χ* ^2^ versus normal, ^b^ *P* < .0001*χ* ^2^ versus LSIL,

^c^ *P* = .0107  *χ* ^2^ versus normal, ^d^ *P* = .0043*χ* ^2^ versus LSIL,

^e^ *P* = .0217*χ* ^2^ versus normal, ^f^ *P* = .0422*χ* ^2^ versus LSIL.

**Table 9:** HPV status and frequency of Fas promoter -670 polymorphism in exfoliated cervical cell samples.

Study group	*n*	Genotype at Fas promoter -670		OR	95% CI	*P*f
		AA	GA + GG			
High-risk HPV−						
Normal	53	15 (28.3%)	38 (71.7%)	1		
LSIL	121	36 (29.8%)	85 (70.2%)	0.93	0.44–1.95	.847
HSIL	9	1 (11.1%)	8 (88.9%)	3.16	0.40–25.04	.276

High-risk HPV+						
Normal	10	4 (40.0%)	6 (60.0%)	1		
LSIL	46	15 (32.6%)	31 (67.4%)	1.38	0.34–5.66	.655
HSIL	40	4 (10.0%)	36 (90.0%)	6.00	1.32–27.37	.021

OR: odds ratio.

CI: confidence interval.
